# Tidal control of the flow through long, narrow straits: a modeling study for the Seto Inland Sea

**DOI:** 10.1038/s41598-019-47090-y

**Published:** 2019-08-29

**Authors:** Masao Kurogi, Hiroyasu Hasumi

**Affiliations:** 1 Japan Agency for Marine-Earth Science and Technology (JAMSTEC), Yokohama, Japan; 2 Atmosphere and Ocean Research Institute, The University of Tokyo, Kashiwa, Japan

**Keywords:** Physical oceanography, Fluid dynamics

## Abstract

Even in coastal oceans where tidal currents are predominant, long-term mean currents are of great interest since they are responsible for the transport of materials over long timescales. Tides could significantly affect mean currents in long, narrow straits due to tide-topography interaction, but it is yet unclear how and to what extent tides control throughflows. Here, we focus on the throughflow in the Seto Inland Sea, Japan, which has enormous impacts on the marine environment while its long-term mean characteristics, even the flow direction, are not well described by observations. By using a state-of-the-art ocean model, we show that the simulated throughflow is eastward on annual average and its volume transport is considerably suppressed by tides. It is found that tides enhance mixing and induce time-mean eddies, and both work to reduce the throughflow. A westward throughflow was previously estimated based on an acoustic measurement. The discrepancy between this estimate and our result would be due to whether or not such eddies are taken into account. These findings imply that tides may also suppress the throughflow of the other straits around the world. Revealing such tidal effects may contribute to a better performance of oceanic and climate simulations.

## Introduction

Coastal seas, especially closed ones are susceptible to human activities. Conversely, human activities such as coastal fisheries and aquaculture are also affected by changes in the environment of coastal seas. Understanding long-term mean currents which are responsible for the transport of materials over long timescale is essential for considering the preservation of the marine environment. However, for most coastal seas including the Seto Inland Sea (SIS) which is the target region of this study, there are not enough observations for such long-term mean currents.

**Figure 1:**
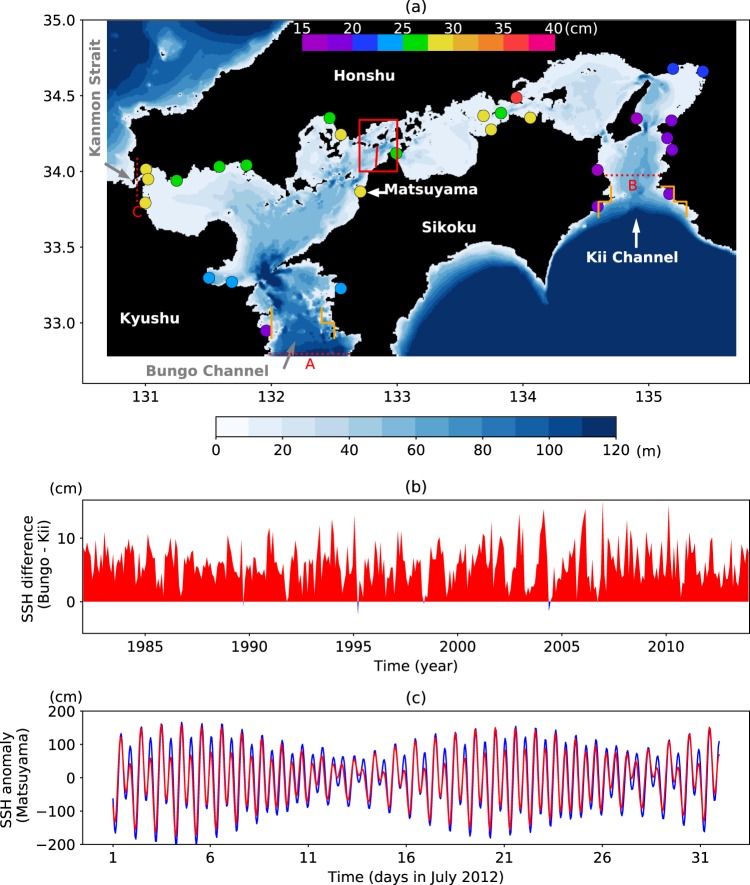
(**a**) Domain of the innermost (L3) model. Blue shading indicates the bottom topography. Filled circles denote root-mean-square errors of hourly SSHA during July 2012. (**b**) SSH difference between the Bungo and Kii Channels calculated from the reanalysis data (FORA-WNP30^6^). SSH of these channels is estimated by averaging values on the coast indicated by the orange lines in (**a**). (**c**) SSHA for observations of tide gauge data (blue) and TIDE (red) at Matsuyama. Observed sea level data used in (**a**,**c**) are download from the JODC website. This figure was prepared with Matplotlib^27^ (version 2.2.2) package in Anaconda (version 5.2.0, https://www.anaconda.com/).

SIS located in the western part of Japan is a semi-enclosed coastal sea surrounded by three Japanese islands (Honshu, Kyushu, and Shikoku, see Fig. 1a). SIS contains many islands and the coastal geometries are complicated. SIS is one of the most industrialized regions in Japan, and serious environmental issues such as red tides have occurred so far. There are many studies concerning the environment of SIS. For these studies and the historical changes in the environment of SIS, the reader is referred to a review^1^.

Water in SIS is exchanged with the Pacific Ocean through the Bungo and Kii Channels and with the Japan Sea through the Kanmon Strait (Fig. 1a). This water exchange has a significant influence on the environment of SIS by forming the flow through SIS (throughflow). Though tidal currents are strong in SIS, currents with longer timescales than the principal diurnal or semidiurnal tide control the long-term mean water properties in SIS. However, even the direction of the throughflow on such longer timescales is not well known. According to the first direct measurement^2^ of the net volume transport of the throughflow in SIS using reciprocal sound transmissions, the net westward volume transport was estimated to be about 1.3 × 10^4^ m^3^ s^−1^ on average for six months of 2012.

Previous studies^3,4^ suggested that the sea level difference between the Bungo and Kii Channels drive the throughflow. Based on the same data of the above direct measurement^2^, it is also pointed out that the sea level difference causes variation in the volume transport of the throughflow^5^. Figure 1b shows the sea level difference estimated from the sea surface height (SSH) of high-resolution reanalysis data (FORA-WNP30^6^). According to this data, the monthly average SSH is higher in the Bungo Channel than in the Kii Channel over almost the entire data period. Therefore, an eastward volume transport is expected if the SSH difference between the channels drives the throughflow, which is opposite to the observational estimate^2^. The throughflow direction in previous numerical simulations^7,8^ using ocean general circulation models (OGCMs) with realistic settings is also eastward, consistent with the SSH difference between the channels during the corresponding periods.

The effects of strong tides in SIS on its throughflow have not well been investigated. Because the sporadic intrusion of Kuroshio water in the Bungo Channel is pointed out to be blocked during spring tides due to tidal mixing and tidal residual eddies^9,10^, it is expected that tides may similarly suppress the throughflow. Taking this into account, a recent numerical simulation^8^ used enhanced horizontal viscosity near the coast for better representation of currents by suppressing the throughflow. It should also be noted that an assumption used by the observational study^2^, that time-averaged currents are parallel to the tidal currents, is not appropriate in the presence of tidal residual eddies. The existence of such eddies may account for the discrepancy in the direction of the throughflow between the above analysis based on the SSH difference and the result of the observational study^2^.

In this paper, the effects of tides on the SIS throughflow are investigated through a series of experiments with and without tides using a state-of-the-art OGCM. Further, the simulated results are compared to the observational study^2^ focusing on tidal residual currents.

## Results

### Model performance of representing tides

Two basic experiments are executed to investigate the effects of tides on the throughflow. The one is NTIDE in which tides are not included and the other is TIDE in which tides are explicitly represented. Before showing detailed analyses, the performance of representing tides in TIDE is examined. The simulated SSH basically represents the observed variation of sea level at tidal stations. The root-mean-square difference of the hourly SSH anomaly (SSHA) between the simulation and observations at tidal stations (Fig. 1a) is relatively small (15–25 cm) near the southern openings of SIS and tends to be large (20–40 cm) in the inner part of SIS. Figure 1c shows SSHA at Matsuyama (Fig. 1a), which is near the observational section^2^ (red line in Fig. 1a). Though the RMS error of SSHA at this station is relatively large, the simulated amplitude, phase, and modulation between spring and neap tides are reasonably represented and are thought to be suitable for use in the analyses of this study.

### Net volume transport of the throughflow

**Figure 2:**
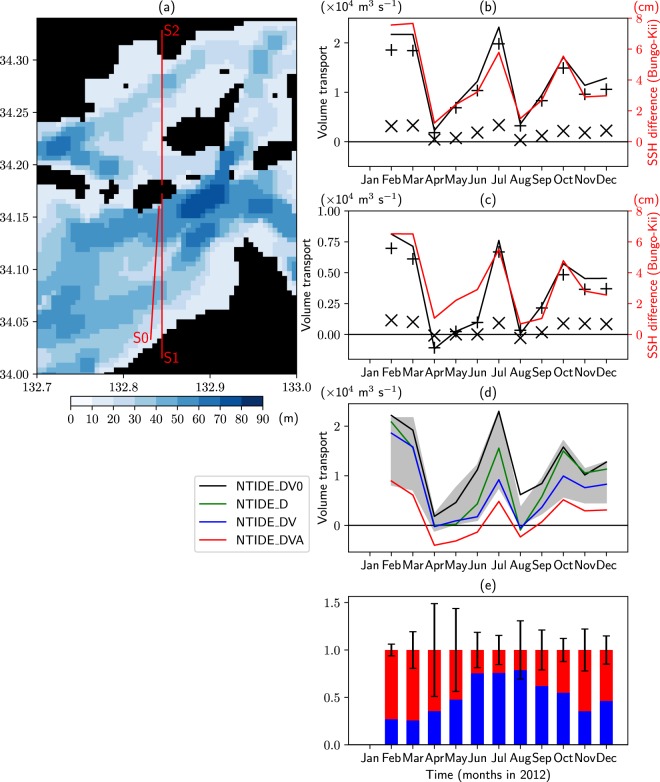
(**a**) Bottom topography in the region inside the red rectangle in Fig. 1a. S0 is the observational section^2^. (**b**) Monthly eastward volume transport across sections S1 (+) and S2 (×) in (**a**), their sum (black line), and the SSH difference between the Bungo and Kii Channels (red line) for NTIDE. SSH of the Bungo (Kii) Channel is calculated by averaging SSH values next to the coast between 32.9°N and 33.1°N (33.7°N and 33.9°N). Barometric pressure correction is applied in calculating SSH. (**c**) Same as (**b**) but for TIDE. (**d**) Monthly eastward volume transport across sections S1 and S2 in (**a**) for TIDE (lower bound of shading), NTIDE (upper bound of shading), and additional experiments (lines). (**e**) Contribution of enhanced vertical mixing by tides (blue) and tidal residual eddies (red) to the reduction of the volume transport of the throughflow. The former (latter) is estimated by the difference of the volume transport between NTIDE_DV0 and NTIDE_DV (NTIDE_DV and NTIDE_DVA). The error bars are drawn based on the larger magnitude of the differences of the volume transport between NTIDE_DV0 and NTIDE, and between TIDE and NTIDE_DVA. These values are normalized by the difference of the volume transport between NTIDE_DV0 and NTIDE_DVA. This figure was prepared with Matplotlib^27^ (version 2.2.2) package in Anaconda (version 5.2.0, https://www.anaconda.com/).

The net volume transport through SIS is calculated at sections S1 and S2 in Fig. 2a. These sections are chosen for accurate calculation of volume transport since the velocity points of the model are located along them. The volume transport in NTIDE (Fig. 2b) is eastward and almost accounted for by that at section S1, which is near the observational section S0 (Fig. 2a). The net transport is mostly controlled by the balance between the flow through the Kii Channel and the sum of the flow through the Bungo Channel and the Kanmon Strait (not shown). The variation of the net volume transport is almost in phase with the SSH difference between the Bungo and Kii Channels (Fig. 2b), suggesting that the pressure difference between the channels drives the throughflow, as pointed out by previous studies^3–5^. These characteristics are also the same for TIDE (Fig. 2c). Thus, the simulated eastward throughflow is consistent with the analysis given in the introduction. However, it is opposite to the observational study^2^. The reason for this discrepancy is examined later.

The volume transport of TIDE is different from NTIDE in the following two points. One is that a weak negative value appears in April in spite of the positive SSH difference. This issue is will be discussed later. Another difference is that the volume transport is much smaller in TIDE. This will be examined in the next.

### Effects of tides in reducing volume transport

Since vertical diffusivity and viscosity are enhanced in TIDE compared to NTIDE (Figs S1 and S2), the effects of enhanced mixing are investigated by executing additional experiments. These experiments are the same as NTIDE except that different types of vertical viscosity and diffusivity are used in the innermost model region (Fig. 1a) without the turbulence closure scheme. For NTIDE_DV0, the monthly output of vertical viscosity and diffusivity of NTIDE is used. NTIDE_D (NTIDE_DV) is the same as NTIDE_DV0, but the monthly output of vertical diffusivity (diffusivity and viscosity) of TIDE is used inside SIS. Figure 2d compares the volume transport among these experimental cases. The volume transport of NTIDE_DV0 is mostly the same as NTIDE (upper bound of shades). By using enhanced vertical diffusivity, the volume transport is suppressed considerably in NTIDE_D. Further, the volume transport is suppressed to the level of TIDE (lower bound of shades) from April to September in NTIDE_DV where both enhanced vertical diffusivity and viscosity are used. Therefore, it is concluded that both vertical diffusivity and viscosity enhanced by tides work to reduce the eastward volume transport of the throughflow.

**Figure 3:**
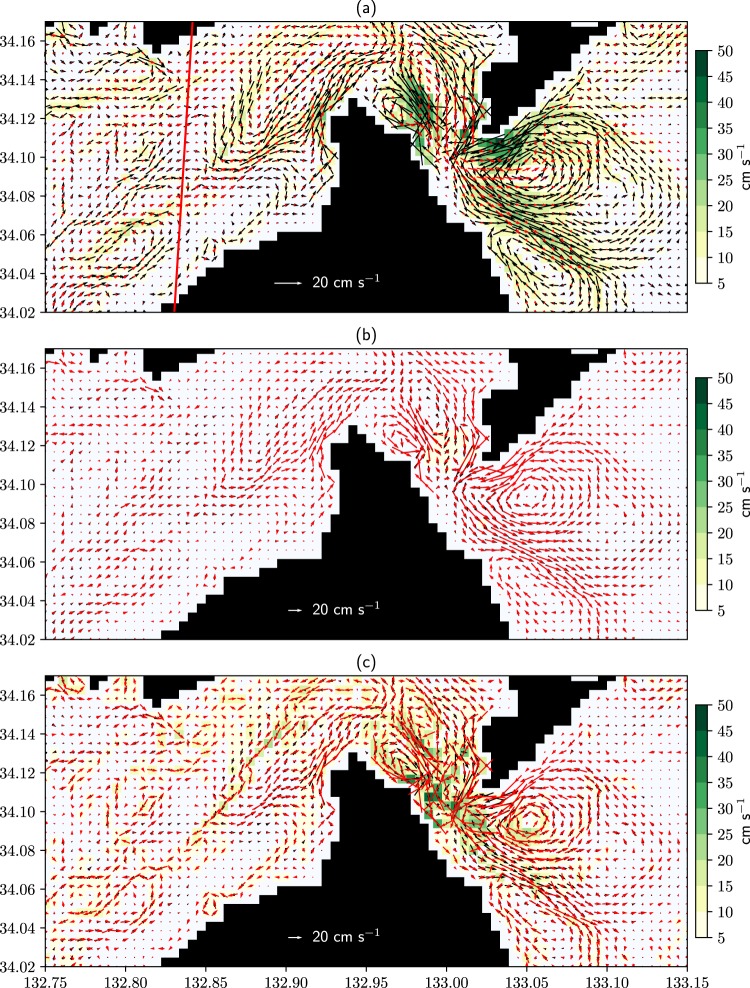
(**a**) Barotropic velocity for TIDE (black arrows) and NTIDE (red arrows) averaged from February to December 2012. (**b**) Same as (**a**) but red arrows are for the inflow-outflow model and time-average is taken during July 2012. (**c**) Same as (**a**) but red arrows are for NTIDE_DVA. Shades indicate the magnitude of the difference between the two velocity fields drawn in each panel. The red line in (**a**) is along the observational section (S0 in Fig. 2a). This figure was prepared with Matplotlib^27^ (version 2.2.2) package in Anaconda (version 5.2.0, https://www.anaconda.com/).

Next, the effects of tidal residual eddies are examined. The time-averaged barotropic velocity in TIDE is much more complicated than in NTIDE (Fig. 3a). For TIDE, the barotropic kinetic energy is predominant over the baroclinic one inside SIS even in the stratified season (Fig. S3), suggesting that the complicated currents in TIDE result from barotropic tidal residual currents. To confirm this, a numerical experiment is executed with a barotropic inflow-outflow model forced only by the barotropic velocity composed of major tidal constituents at the Bungo and Kii Channels (Methods for detail). The red arrows in Fig. 3b show the averaged barotropic velocity during July 2012 for the barotropic inflow-outflow model. It is almost the same as that in TIDE (black arrows). Therefore, the primary factor causing the complicated structure of time-averaged currents is barotropic tidal residual currents.

To investigate the effects of the above tidal residual currents on the volume transport of the throughflow, an additional experiment, NTIDE_DVA, is executed following ref.^10^. This experiment is the same as NTIDE_DV except that the horizontal divergence of the Reynolds stress for TIDE is added to the momentum equations inside SIS to reproduce the tidal residual currents. The resultant structure of time-averaged barotropic currents in NTIDE_DVA is similar to TIDE (Fig. 3c). The volume transport in NTIDE_DVA is further reduced compared with NTIDE_DV (Fig. 2d). However, it is smaller compared with TIDE (Fig. 2d). This difference may be due to the difference in tidal residual currents: the structure of eddies is somewhat different between NTIDE_DVA and TIDE and the strength of eddies is generally stronger in the former. Regardless of such differences, it is suggested that tidal residual eddies work to reduce the eastward volume transport of the throughflow.

Figure 2e shows the contribution of enhanced vertical mixing due to tides (blue) and tidal residual eddies (red) to the reduction of the volume transport, estimated from the volume transport of NTIDE_DV0, NTIDE_DV, and NTIDE_DVA. Since this estimate assumes that volume transport of NTIDE (TIDE) is equal to NTIDE_DV0 (NTIDE_DVA), the differences between them are considered as an error (error bar of Fig. 2e). Though the estimate is rough due to the relatively large error, there is a seasonal variability: the contribution of enhanced vertical mixing (tidal residual eddies) is relatively large in summer (winter) and small in winter (summer). The greater contribution of the enhanced mixing in summer is thought to be related to a remarkable difference of stratification depending on the presence of the enhanced mixing. In winter, stratification is weak regardless of the presence of enhanced mixing.

### Comparison with the observational study

**Figure 4:**
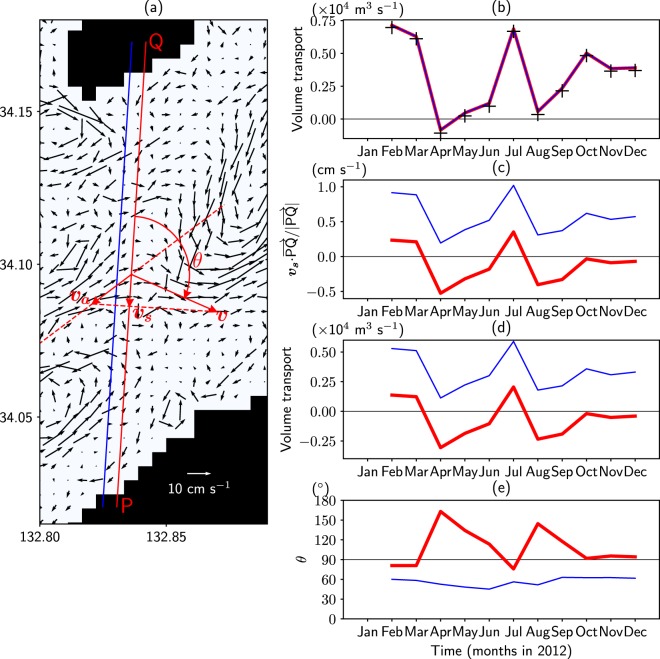
(**a**) Barotropic velocity for TIDE (black arrows) averaged from February to December 2012. Section PQ is along the observational section (S0 in Fig. 2a). The red arrows schematically show velocity ***v*** averaged along PQ, the along-section component of velocity ***v***_*s*_ and the converted velocity ***v***_*a*_ along the dotted line. The red lines in (**b**–**e**) are monthly means of volume transport across section PQ, ${{\boldsymbol{v}}}_{{\rm{s}}}\cdot \overrightarrow{{\rm{PQ}}}/|\overrightarrow{{\rm{PQ}}}|$, eastward volume transport estimated in a similar manner by the observational study^2^, and current direction (θ in (**a**)), respectively. Quantities in (**c**–**e**) are calculated by using barotropic velocity averaged along PQ. The marker (+) in (**b**) shows monthly eastward volume transport across section S1 (Fig. 2a). The blue lines in (**b**–**e**) are values when a slightly westward section (blue lines of (**a**)) is selected. This figure was prepared with Matplotlib^27^ (version 2.2.2) package in Anaconda (version 5.2.0, https://www.anaconda.com/).

In this subsection, the reason for the discrepancy in the throughflow direction between TIDE (eastward) and the observational study^2^ (westward) is examined, focusing on tidal residual currents. Because the velocity points of the model are not located along the observational section, quantities along the observational section are approximated by using interpolated values along PQ in Fig. 4a. Figure 4b compares volume transport along the section (red line) calculated by using such interpolated values and the accurate volume transport (+) along the section S1 (Fig. 2a). Since the former is almost the same as the latter, it can be said such a method of approximation is sufficiently valid.

In the acoustic measurement of the observational study^2^, the velocity component along the section (***v***_*s*_ in Fig. 4a) is obtained from the reciprocal travel time data. To estimate volume transport from ***v***_*s*_, the direction of the currents is necessary. In the observational study^2^, this direction was estimated to be along the dotted line in Fig. 4a based on tidal currents. Then, ***v***_*s*_ is converted to current velocity (***v***_*a*_ Fig. 4a) to estimate the time-averaged volume transport.

Figure 4c shows ${{\boldsymbol{v}}}_{{\rm{s}}}\cdot \overrightarrow{{\rm{PQ}}}/|\overrightarrow{{\rm{PQ}}}|$ calculated by averaging the barotropic velocity along PQ in TIDE. As shown in this figure, ***v***_*s*_ is southward on average for the observed period of 6 months (from April to October 2012 excluding August). This direction is consistent with the observed direction because the westward throughflow in the observational study^2^ means that southward ***v***_*s*_ is observed. If the volume transport in TIDE is evaluated as in the observational study^2^ (by converting ***v***_*s*_ to ***v***_*a*_ in Fig. 4a), it becomes westward on average (Fig. 4d) as in that study^2^, though the amount is much smaller. An increasing trend of the eastward volume transport from April to July, with the throughflow direction changing from westward to eastward, is also consistent with that study^2^ (see Fig. 9 of Zhang *et al*.^2^). These results mean that the along-section component of velocity (directly observed quantity) is qualitatively consistent between the observational study^2^ and TIDE.

The difference in the throughflow direction between the observational study^2^ and TIDE results from the different direction of the time-averaged currents. The observational study^2^ used the current direction estimated from tidal currents (the dotted line in Fig. 4a). Though the predominant direction of tidal currents in TIDE is roughly parallel to this direction (not shown), the horizontal structure of time-averaged barotropic currents is complicated, as shown in Fig. 4a. The angle θ (Fig. 4a) between section PQ and the simulated barotropic velocity averaged along the section (***v*** in Fig. 4a) exceeds 90° (Fig. 4e). If 90° < θ < 180° as in the case of TIDE, an estimation of currents by assuming the direction as the dotted line in Fig. 4a results in incorrect westward currents (***v***_*a*_ in Fig. 4a), opposite to the correct eastward currents (***v*** in Fig. 4a). The above results suggest that the tidal residual eddies, which are not taken into account by the observational study^2^, may be responsible for the discrepancy in the throughflow direction between the observational study^2^ and TIDE.

There are some limitations with respect to the accuracy of the above comparison. The blue lines in Fig. 4b–e show values of each quantity when a slightly westward section (blue line in Fig. 4a) is selected. The net eastward transport is almost the same as that for PQ (Fig. 4b). However, the quantities in Fig. 4c–e, which are calculated by using barotropic velocity averaged along the section, is much different from those for PQ. The drastic change of these quantities occurs because barotropic velocity averaged along the section is sensitive to the position of the section relative to small-scale tidal residual eddies. Because tidal residual eddies are generated by the interaction between tides and topographies, the quantities may be sensitive to slight differences of the model topography from real one. For a more accurate comparison, higher resolution experiments based on more precise topography data are required. Nevertheless, it can still be concluded that tidal residual eddies are related to the discrepancy in throughflow direction between the observational study^2^ and TIDE.

## Summary and Discussion

In the present study, the effects of tides on the throughflow of SIS are investigated by executing numerical simulations with and without tides. In both cases, the simulated throughflow is eastward on average. The volume transport of the throughflow is in phase with the difference in SSH between the Bungo and Kii Channels, suggesting that the throughflow is driven by the pressure difference between the channels. The volume transport of throughflow is considerably more suppressed in the case with tides than in the case without tides. A series of additional experiments are then conducted focusing on the following effects of tides: enhancement of vertical diffusivity and viscosity and generation of tidal residual eddies. It is found that all of them work to reduce the eastward volume transport of the throughflow.

From the comparison between our simulation and the observational study^2^, it is found that the along-section component of velocity (directly observed quantity) is qualitatively consistent, but the direction of the throughflow is opposite. The difference arises from the choice of the direction of time-averaged currents by the observational study^2^, in which it is assumed to be the same as the direction of tidal currents. However, our simulation indicates that the direction of time-averaged currents is considerably different from that of tidal currents due to the existence of tidal residual eddies.

Here, the issue of westward volume transport appearing in April in TIDE (Fig. 2c) is discussed. Because the SSH difference is relatively small in April, other factors may drive the throughflow westward. Candidates of such factors are wind stress^11,12^, the spatial variation of density^4^, and tidal residual currents^13^ (eddies). To investigate the effects of wind stress, an additional experiment TIDE_NW is executed. This experiment is the same as TIDE but wind stress is not imposed in the innermost model. Though the SSH difference and volume transport in TIDE_NW are somewhat different from TIDE, westward volume transport appears in spite of the positive SSH difference in April as in TIDE (Fig. S4). This means that westward volume transport in April is not caused by wind stress. The spatial variation of density is also unlikely to be the cause of the westward volume transport as follows. The near-surface potential density in April tends to be large in the western region with about 0.25 kgm^−3^ larger in the Bungo Channel than in the Kii Channel (Fig. S5). Such horizontal variation of density will work to drive the throughflow eastward. The tidal residual currents may be the cause of the westward volume transport. In the previous study^13^ of tidal simulation, the southward tidal residual currents appear at the Bungo Channel at spring tide, which means the existence of westward tidal residual currents through the SIS. The westward volume transport appearing in some months in NTIDE_DVA (Fig. 2c), which includes terms to reproduce the tidal residual currents, is also consistent with the existence of westward tidal residual currents. Further study is needed to elucidate the effects of tidal residual currents.

The detailed mechanisms of how tides suppress the throughflow need further investigation, and the issues to be addressed are summarized below. During the course along the throughflow, a fluid should lose a similar amount of momentum to what is received from the pressure gradient caused by the pressure difference between the channels; otherwise, unrealistic acceleration would occur. This loss of momentum occurs at solid boundaries partly due to viscosity (friction) and partly due to form drag. Enhanced vertical diffusivity and viscosity may contribute to the efficient momentum transfer toward the deep layer and loss due to bottom topography by the bottom friction and form drag. Tidal residual eddies may enhance the horizontal momentum transfer and loss at lateral boundaries due to viscosity and form drag. These eddies may also contribute to efficient loss of momentum by making the streamline complicated in a manner that enhances form drag at lateral boundaries. In this way, a smaller volume transport may be enough to lose momentum received from the pressure gradient for the experimental case with tides.

Another point to be addressed is the dependency of the volume transport of the throughflow on resolution. The horizontal resolution of this study (about 500 m) may be insufficient to represent small-scale tidal residual eddies in SIS as suggested by the previous study^9,10^ which showed that a horizontal mesh of 1/200°−1/100° is still insufficient to represent such eddies in the Bungo Channel. We are planning to execute simulations with a horizontal mesh of about 1/1000° in the future.

The results of this study suggest that tides may also suppress the throughflow of the straits in other parts of the world’s oceans. In the North Pacific, examples of straits whose throughflow may be profoundly affected by tides are the Tsushima, Tsugaru, Soya, and Bering Straits. Revealing the effects of tides on throughflows of such straits may contribute to a better representation of oceanic conditions in OGCMs and climate simulations.

## Methods

### A nested-grid OGCM

The model used in this study is a two-way nested-grid OGCM based on an ice-ocean coupled model named COCO^14^. The nesting method is almost the same as that in ref.^15^ with some improvements in conservation and sea-ice coupling. The model consists of four sub-models. The outermost sub-model (hereafter, L0) is a global OGCM based on the tripolar grid^16^ with a horizontal resolution of 1/4° × 1/4°cosϕ for the spherical coordinate region south of 63°N, where ϕ is latitude. To this model, the following three regional sub-models, L1, L2, and L3 based on the spherical coordinate system, are nested. L1, L2, and L3 cover the western North Pacific (105°E–175°W, 17.23°N–62.89°N), the western part of Japan (128.25°E–138.25°E, 31.95°N–36.09°N), and SIS (Fig. 1a), respectively. The horizontal resolution of L1, L2, and L3 is 1/12° × 1/12°cosϕ, 1/60° × 1/60°cosϕ, and 1/180° × 1/180°cosϕ, respectively. For each sub-model, there are 62 vertical levels. The bottom topographies of L0, L1, and L2 are based on ETOPO1^17^. The 500 m gridded bathymetry data of the Japan Oceanographic Data Center (JODC, http://www.jodc.go.jp/jodcweb/index.html) and the Shuttle Radar Topographic Mission data^18^ are used to create the bottom topography of L3. There is no explicit horizontal diffusivity. For horizontal viscosity, the biharmonic version of Smagorinsky viscosity^19^ is used. Vertical viscosity and diffusivity are based on the turbulence closure of ref.^20^.

The model is integrated as follows. Firstly, a nested-grid OGCM composed of L0 and L1 is spun up from year 2000 to 2012. Then, by interpolating the results into L2, a triply nested-grid OGCM (L0, L1, and L2) is integrated for 1 day, January 1, 2012. Further, by interpolating the results into L3, the quadruply nested-grid OGCM (L0, L1, L2, and L3) is integrated through the end of 2012. The models are driven by the atmospheric boundary condition data and the river runoff data of the early version of JRA55-do^21,22^. In the region east of 116.9°E of L1, potential temperature and salinity are restored to a high-resolution reanalysis dataset^6^ with the time constant of 1 day.

When integrating the quadruply nested-grid OGCM, two basic experimental cases are executed. In the first experiment, TIDE, tides are explicitly represented by including the tidal forcing and dissipation terms in the barotropic momentum equations. The tidal forcing is calculated from the positions of astronomical bodies^23^ (the sun, earth, and moon). The parameterization^24^ of the tidal dissipation processes is used for L0 and L1 and applied only for the deviation from the 25-hour running mean of barotropic velocity^25^. The second experimental case is NTIDE in which the tidal forcing is not included.

Additional experiments are executed to investigate effects of tide-induced enhanced vertical mixing on the volume transport of the throughflow. These experiments are the same as NTIDE except that different types of vertical viscosity and diffusivity are used in the L3 model region without the turbulence closure scheme. For NTIDE_DV0, the monthly output of vertical viscosity and diffusivity of NTIDE is used. NTIDE_D (NTIDE_DV) is the same as NTIDE_DV0, but the monthly output of vertical diffusivity (diffusivity and viscosity) of TIDE is used inside the dotted lines A, B, and C in Fig. 1a.

Another additional experiment NTIDE_DVA is executed following ref.^10^ to investigate the effects of the tidal residual currents on the volume transport of the throughflow. This experiment is the same as NTIDE_DV except that the following term,

1 $$(\overline{{u^{\prime} }_{{\boldsymbol{BT}}}\frac{\partial {u^{\prime} }_{{\boldsymbol{BT}}}}{\partial x}+{v^{\prime} }_{{\boldsymbol{BT}}}\frac{\partial {u^{\prime} }_{{\boldsymbol{BT}}}}{\partial y}},\,\overline{{u^{\prime} }_{{\boldsymbol{BT}}}\frac{\partial {v^{\prime} }_{{\boldsymbol{BT}}}}{\partial x}+{v^{\prime} }_{{\boldsymbol{BT}}}\frac{\partial {v^{\prime} }_{{\boldsymbol{BT}}}}{\partial y}})$$

is added to the momentum equations inside SIS (inside dotted lines A, B, and C in Fig. 1a), where the overline indicates time average (from February to December 2012) and (${u^{\prime} }_{{\boldsymbol{BT}}}$, ${v^{\prime} }_{{\boldsymbol{BT}}}$) is the deviation of horizontal barotropic velocity from the time average in TIDE. This term expresses vorticity transfer from tides to tidal residual currents^26^. It is expected that the basic features of tidal residual currents are reproduced by adding this term to the momentum equations.

Further, to investigate the effects of wind stress, an additional experiment TIDE_NW is executed. This experiment is the same as TIDE except that no wind stress is imposed on momentum equations in the innermost model (Fig. 1a).

### Barotropic inflow-outflow model

To confirm that the complicated time-avaregaed currents in TIDE result from barotropic tidal residual currents, following numerical experiment is executed with a barotropic inflow-outflow model based on COCO^11^. The basin of this model is the same as the innermost model (L3) of a nested-grid OGCM except that the region outside dotted lines A, B, and C in Fig. 1a is not considered. The horizontal and vertical grids are also the same as L3. The model is forced only by the inflow-outflow boundary condition at dotted lines A and B in Fig. 1a. Barotropic velocity composed of four major tidal constituents (M2, S2, O1, K1) calculated from every 10 minutes output of TIDE is used as the boundary condition. The density of water is set to be constant. For horizontal viscosity, the same scheme is used as in TIDE. For vertical diffusivity, the monthly mean (July 2012) of TIDE is used. The model is integrated from June 15 to August 1, 2012.

## Supplementary information

Supplementary Information

## Data Availability

The model output data used to draw figures are available at https://doi.org/10.5281/zenodo.1471095.
