# Supplementary material for: Tidal control of the flow through long, narrow straits: a modeling study for the Seto Inland Sea
Source: Sci Rep. 2019 Aug 29;9:11077. doi: 10.1038/s41598-019-47090-y (PMC6715803; doi:10.1038/s41598-019-47090-y)
Supplement: Supplementary file 1 — Supplementary Information [file 41598_2019_47090_MOESM1_ESM.docx]

**Supplementary Information**

**Tidal control of the flow through long, narrow straits: a modeling study for the Seto Inland Sea**

### Masao Kurogi^1^ and Hiroyasu Hasumi^2^

^1^Japan Agency for Marine-Earth Science and Technology (JAMSTEC), Yokohama, Japan

^2^Atmosphere and Ocean Research Institute, The University of Tokyo, Kashiwa, Japan

Corresponding author

Masao Kurogi

Japan Agency for Marine-Earth Science and Technology (JAMSTEC)

3173-25 Showa-machi, Kanazawa-ku, Yokohama, Kanagawa 236-0001, Japan

E-mail: m_kurogi@jamstec.go.jp

**
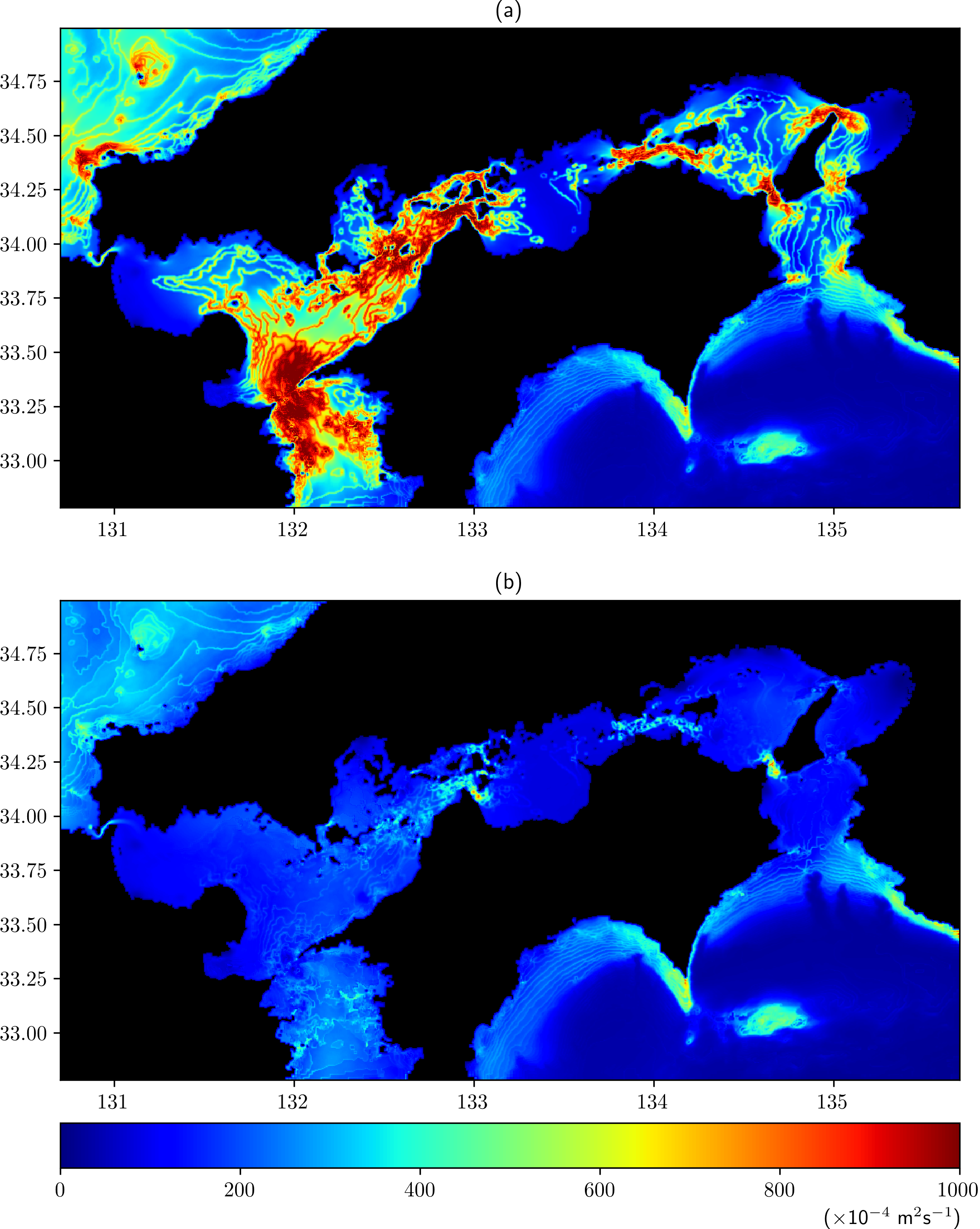
**

**Figure S1.** Vertical average of vertical diffusivity for (a) TIDE and (b) NTIDE. Time-averaged values from February to December 2012 are shown. This figure was prepared with Matplotlib^27^ (version 2.2.2) package in Anaconda (version 5.2.0, https://www.anaconda.com/).


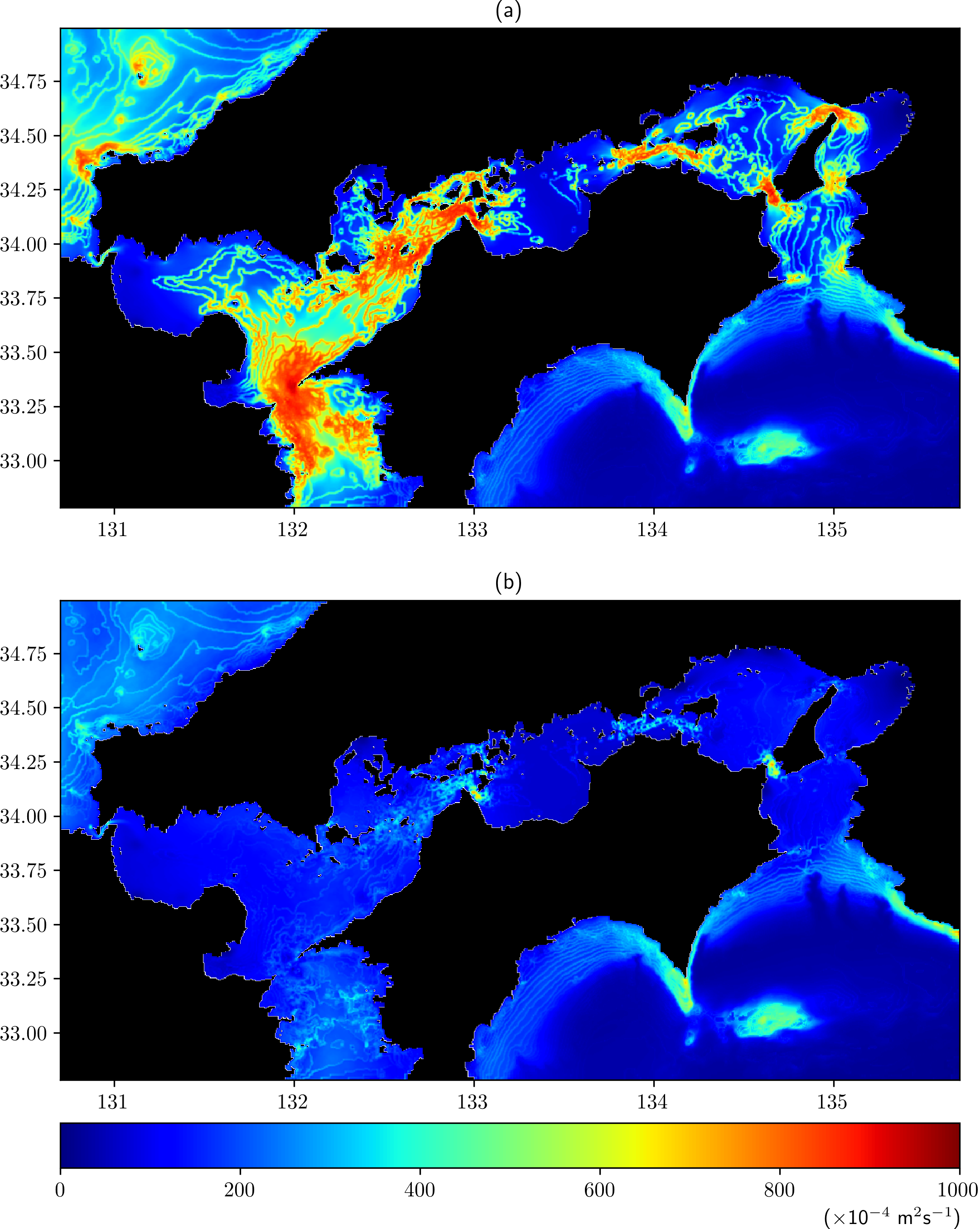


**Figure S2.** Same as Fig. S1, but for vertical viscosity. This figure was prepared with Matplotlib^27^ (version 2.2.2) package in Anaconda (version 5.2.0, https://www.anaconda.com/).


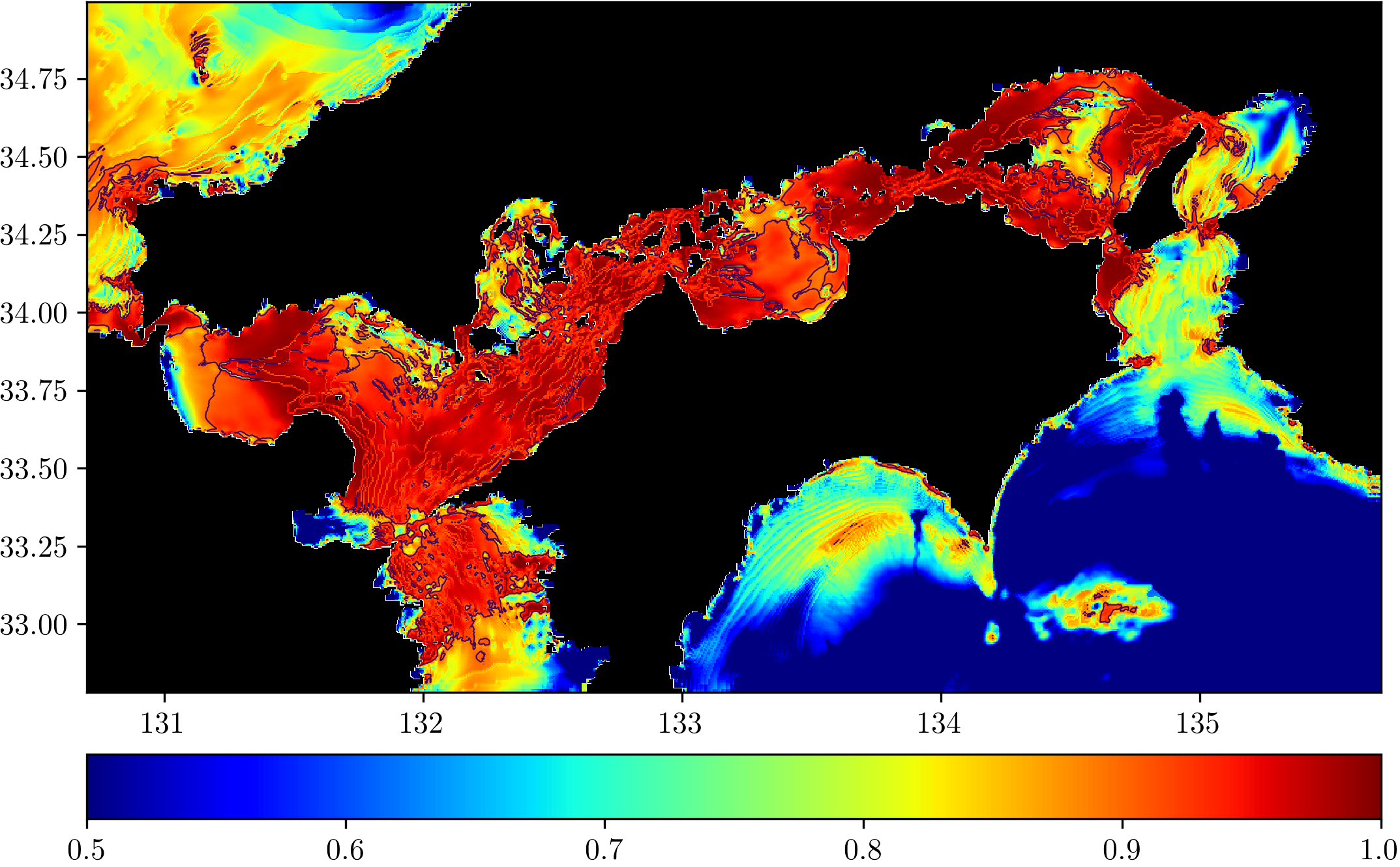


**Figure S3.** Ratio of the barotropic kinetic energy to total one, $\left\langle{\bar{\mathbf{u}}}^{2} \right\rangle/\left\langle\bar{\mathbf{u}^{2}} \right\rangle$, where $\mathbf{u}$, overline, and brackets indicate horizontal velocity vector, vertical average, and time average during July 2012, respectively. Data of velocity averaged every 2 hours are used in the calculation. Contour lines indicate a value of 0.9. This figure was prepared with Matplotlib^27^ (version 2.2.2) package in Anaconda (version 5.2.0, https://www.anaconda.com/).


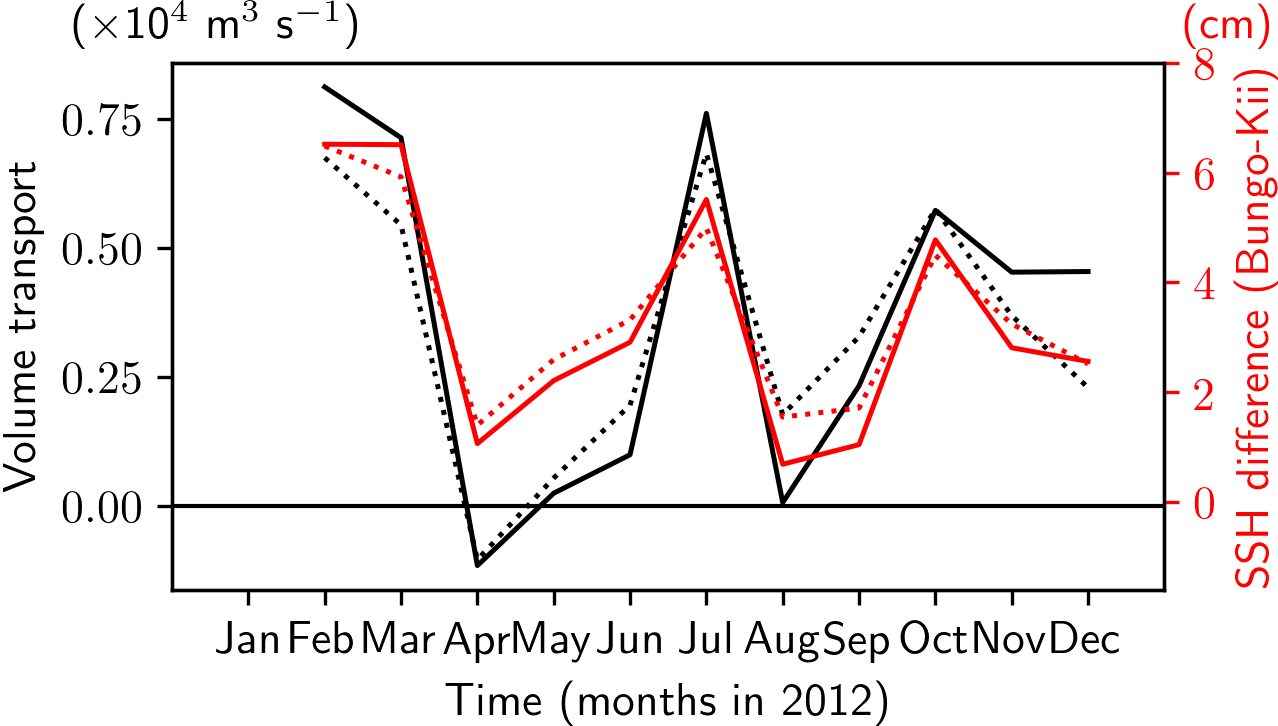


**Figure S4.** Same as Fig. 2c for the solid lines. Dotted lines indicate the volume transport (black) and SSH difference (red) for TIDE_NW. This figure was prepared with Matplotlib^27^ (version 2.2.2) package in Anaconda (version 5.2.0, https://www.anaconda.com/).


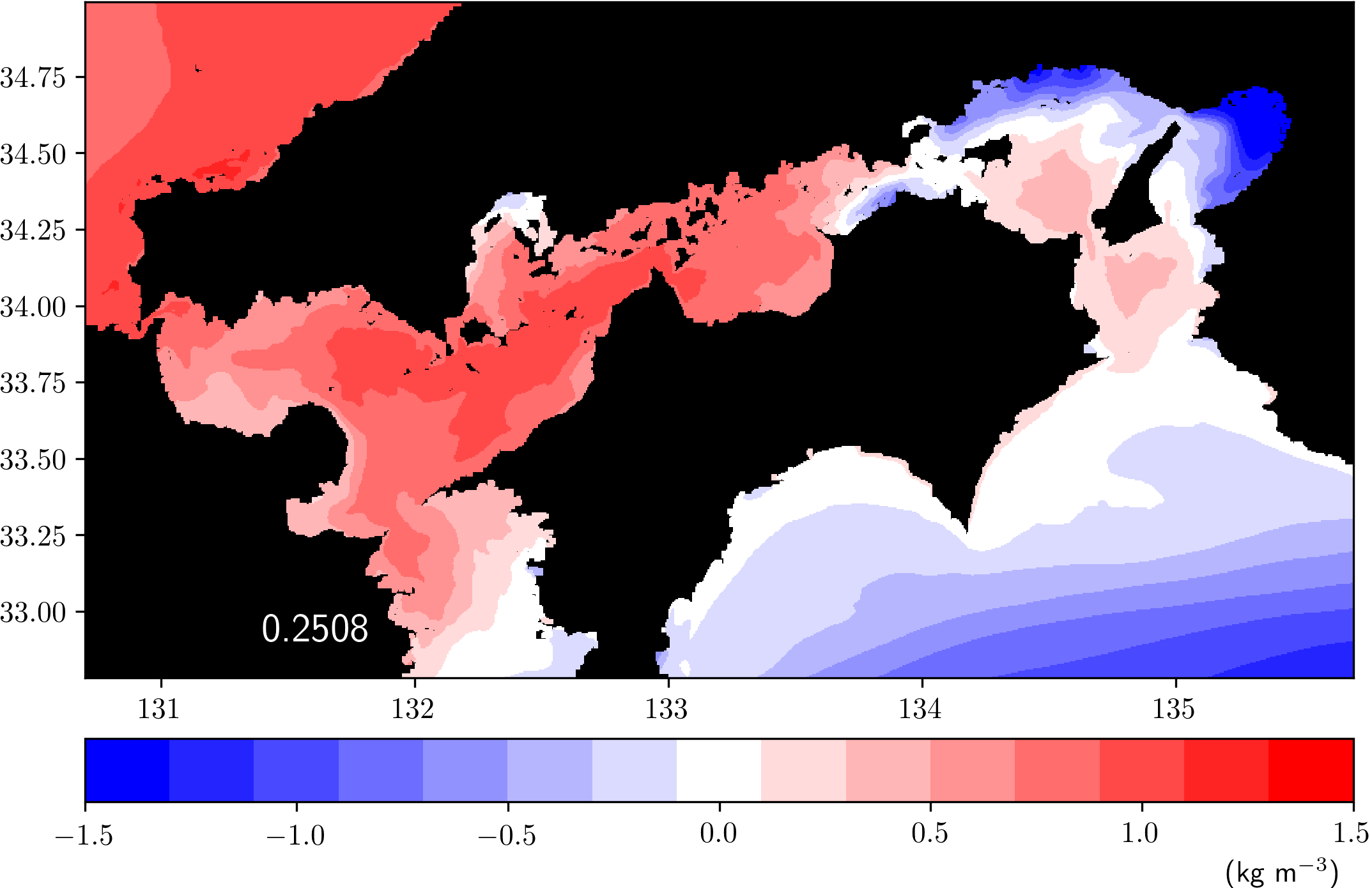


**Figure S5.** Potential density averaged during April and for upper 18m (the maximum depth the flow can pass through the SIS horizontally). Anomaly from the value of the Kii Channel is shown. The numerics indicate the value of the Bungo Channel. Values of the Bungo (Kii) Channel is calculated in the same way as SSH in Figs 2b-c. This figure was prepared with Matplotlib^27^ (version 2.2.2) package in Anaconda (version 5.2.0, https://www.anaconda.com/).
